# Case series: Chemical pneumonitis caused by exposure of sodium ibandronate powder

**DOI:** 10.3389/fpubh.2025.1523207

**Published:** 2025-02-28

**Authors:** Ting Pan, Guofeng Ma, Jiali Qian

**Affiliations:** Department of Pulmonary and Critical Care Medicine, Regional Medical Center for National Institute of Respiratory Diseases, Sir Run Run Shaw Hospital, School of Medicine, Zhejiang University, Hangzhou, China

**Keywords:** chemical pneumonitis, sodium ibandronate, exposure, steroid treatment, case report

## Abstract

This report presents two cases of chemical pneumonitis caused by the exposure of sodium ibandronate powder, a bisphosphonate used for treating osteoporosis. They are both pharmaceutical workers, and they inhaled the powder after an accidental spill in the lab. They developed similar symptoms, but different imaging features, only one showed fibrotic changes. After the blood test and bronchoscopy examination, they both diagnosed as chemical pneumonitis. Their symptoms improved after treatment with glucocorticoids. Their lung lesions resolved completely after continued treatment. The findings suggest that the importance of appropriate safety measures in environments where pharmaceutical powders may be handled, such as in the pharmaceutical industry. Chemical pneumonitis from inhaled sodium ibandronate can have varying CT appearances and may benefit from glucocorticoids therapy.

## 1 Introduction

Many drugs may cause lung injury which is called chemical pneumonitis. Chemical pneumonitis can manifest in various patterns on CT scans, often affecting both the upper and lower airways and typically presenting as diffuse opacities. It may mimic interstitial lung diseases such as hypersensitivity pneumonitis, organizing pneumonia, eosinophilic pneumonia, and other granulomatous reactions (1). While numerous cases of lung injury due to oral medications have been reported (2), there is a scarcity of data on inhalation-induced injuries such as chemical pneumonitis due to bromine compounds, zinc oxide fumes. Different drugs can exhibit distinct patterns on CT scans, ranging from fibrosis and consolidation to ground-glass opacities. Even the same drugs may cause varying extents of lung injury in different patients. However, there have been no reports of chemical pneumonitis due to sodium ibandronate. Herein, we reported a case series of Chemical Pneumonitis caused by exposure of sodium ibandronate powder. This is the first case series about this drug.

## 2 Case 1

**Figure 1:**
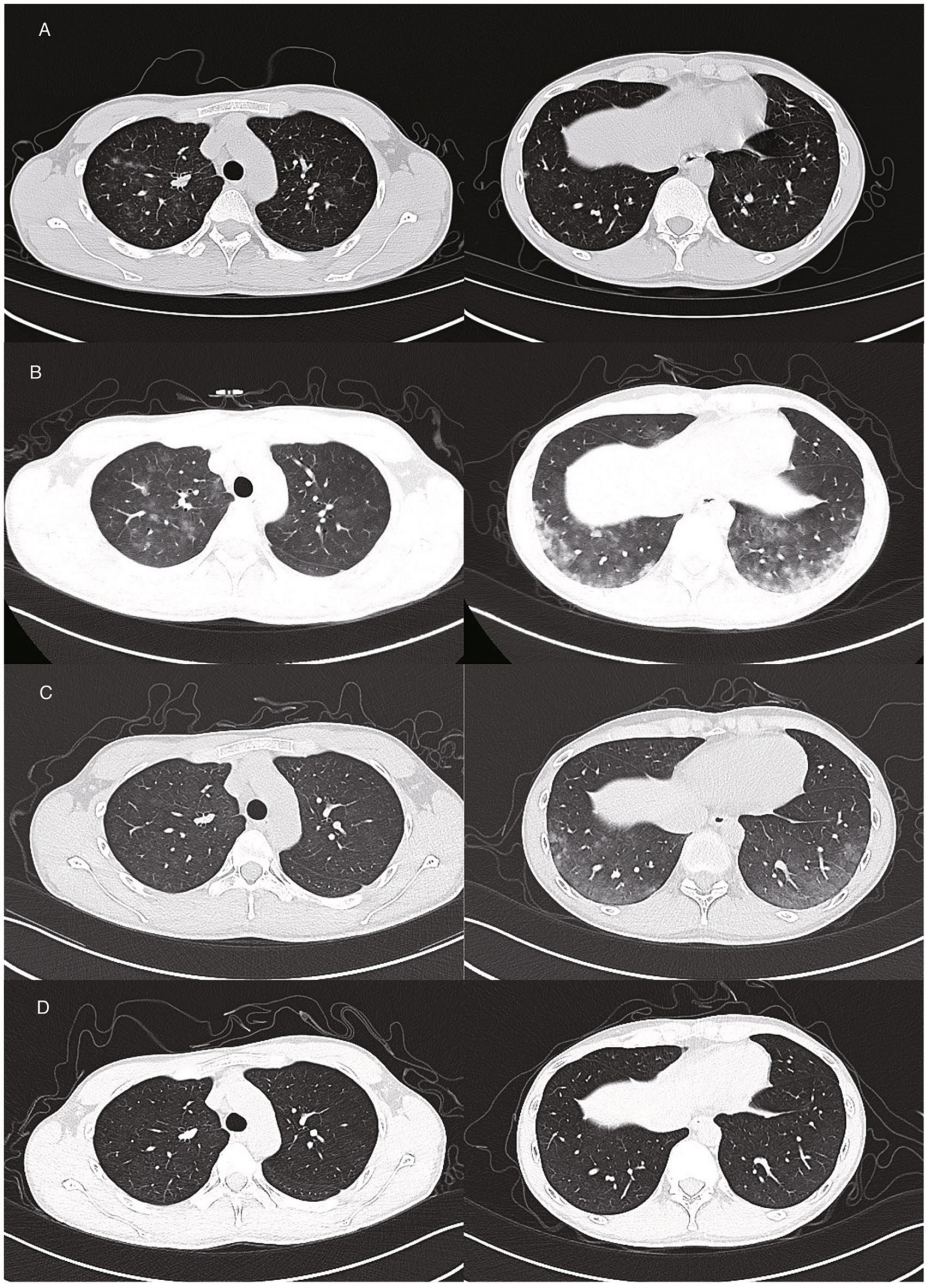
Patient 1: **(A)** Scattered opacities in upper and lower airways on the first day post-exposure. **(B)** Progression of opacities on the third day. **(C)** Partial resolution on the fifth day. **(D)** Complete resolution on the ninth day.

A 26-year-old non-smoker male reported to the emergency department with complaints of headache, fever, and a non-productive cough that had persisted for 24 h. His occupation involved handling sodium ibandronate powder at a pharmaceutical facility, and 2 days ago he had shattered a mass of sodium ibandronate into powder, and put the powder in the capsule, the work last 3 h. He told the doctor he may have inhaled sodium ibandronate powder. Clinical assessment revealed a respiratory rate of 20 breaths per minute, a body temperature of 38°C, and an oxygen saturation of 98% on room air. Auscultation of the lungs disclosed mild crackles bilaterally, and erythematous vesicular lesions were observed on his right arm. Laboratory tests indicated a high neutrophil count, lymphopenia, and increased serum bilirubin levels. Pulmonary function tests showed minor ventilatory impairment. A chest CT scan exposed scattered opacities (Figure 1A). A follow-up scan after 2 days of moxifloxacin therapy showed a marked increase in pulmonary opacities (Figure 1B). Negative results were obtained for microbiological cultures, antinuclear antibodies, and allergen tests. The tests for serum *Mycoplasma pneumoniae*, *Chlamydia pneumoniae* were negative. Respiratory virus panel tests did not detect adenovirus, coronavirus, human metapneumovirus, human rhinovirus/enterovirus, influenza, parainfluenza, respiratory syncytial virus. Bronchoscopic examination revealed generalized erythema and edema of the tracheobronchial mucosa. The bronchoalveolar lavage (BAL) fluid cell count was 20% lymphocytes, 20% neutrophils, and 10% eosinophils, with occasional atypical cells noted, characterized by an enlarged cell body, increased cytoplasm-to-nucleus ratio, and hyperchromatic chromatin with visible nucleoli, and some cell fusion was observed. The specific pathogen was not identified by the next-generation sequencing (NGS) of pathogen from the bronchoalveolar lavage fluid. All the examination shows no evidence about bacteria or virus infection. We suspect that injury may be relative with his working about handling sodium ibandronate powder. The most possible diagnosis is chemical pneumonitis due to sodium ibandronate exposure, and treatment with 40 mg methylprednisolone twice daily was initiated. His symptoms resolved after 8 days of steroid therapy, then go back to home with oral glucocorticoid, In the following visit of outpatient, a significant improvement in imaging was noted (Figures 1C,D). And he told the fact that his factory had checked the environment of his workplace and found the ventilation equipment in chemical fume hood had fault in these days. Even though he wore a mask for protection, he inhaled sodium ibandronate. The factory repaired the ventilation equipment. The fact confirmed the diagnosis of chemical pneumonitis due to sodium ibandronate.

## 3 Case 2

**Figure 2:**
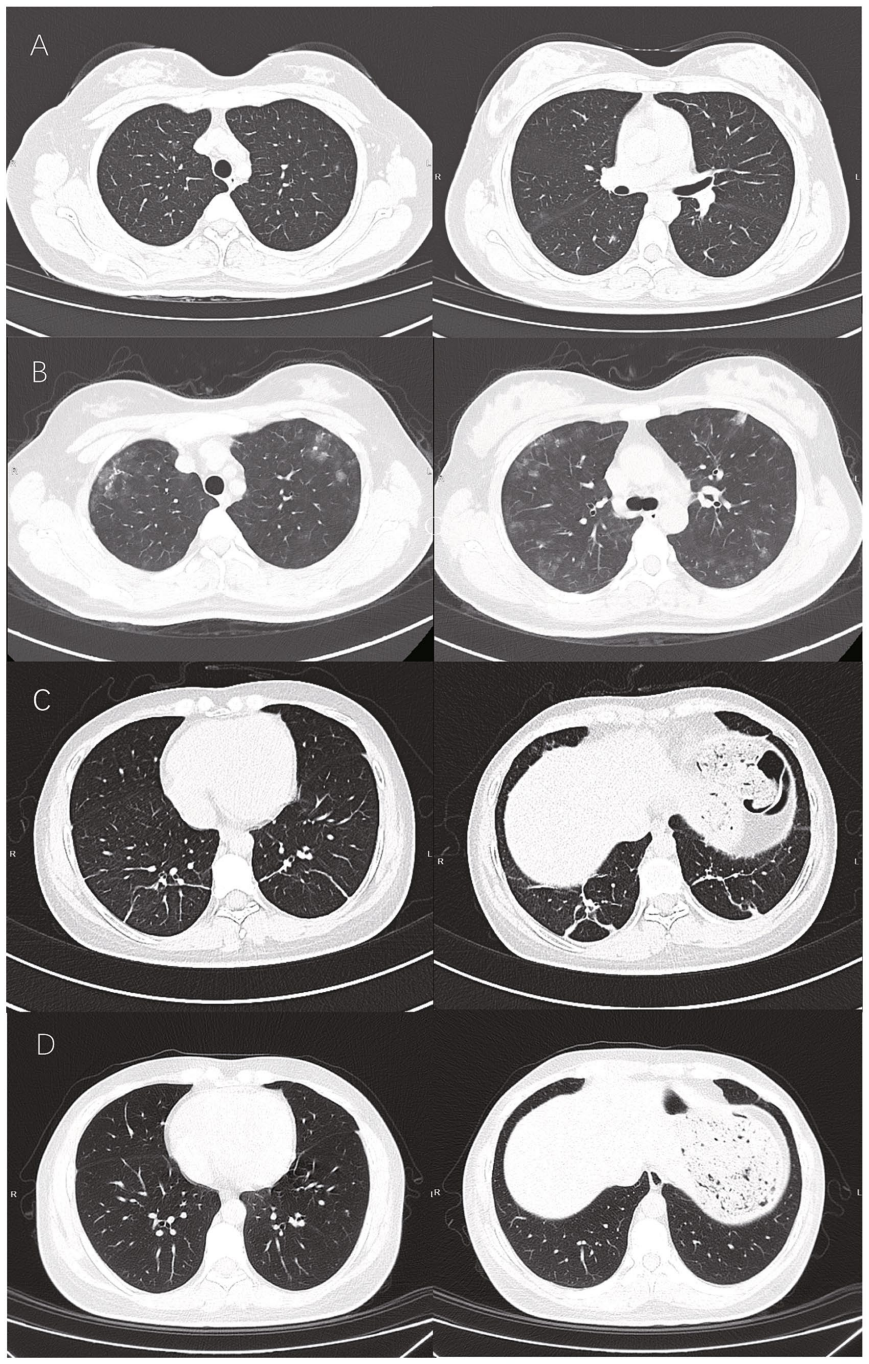
Patient 2: **(A)** Scattered opacities in upper and lower airways on the first day post-exposure. **(B)** Progression on the third day. **(C)** Fibrotic changes in bilateral lower lobes on the eleventh day. **(D)** Complete resolution on the 28th day.

This 25-year-old female patient, a coworker of the first case, was also present during the powder handling workplace, suffered a similar injury. She was a non-smoker and presented with fever and a non-productive cough, seeking medical attention 1 day after the drug exposure incident. Laboratory tests showed leukocytosis and lymphopenia. A chest CT scan showed scattered opacities (Figure 2A). She was initially treated with moxifloxacin for 3 days, after which she developed exertional dyspnea and was admitted to the respiratory medicine department. A subsequent CT scan showed significant progression (Figure 2B). Given the similarity to the first case, she underwent bronchoscopic examination, which was consistent with the findings in case 1. No microbiological evidence was found. The BAL fluid cell composition was 20% lymphocytes, 6% neutrophils, and 1% eosinophils. The NGS of pathogen showed no infection of bacteria, fungi or virus. She was also suspected as chemical pneumonitis. Following corticosteroid therapy, her condition improved. A CT scan taken 9 days after treatment showed some fibrotic changes (Figure 2C), contrasting with case 1. Corticosteroid therapy was continued, and a repeat image after 26 days showed complete resolution of the lesions (Figure 2D).

## 4 Discussion

These two cases show how to find the causes of pneumonitis, we make the blood test, sputum culture and bronchoscopy. We use the metagenomics analyses in BAL fluid sample. Metagenomics analyses allow quick and easy detection and identification of a great variety of pathogen in different clinical samples, particularly for the use of NGS techniques. It is important in differential diagnoses of lung inflammation. Without any evidence of bacteria, fungi or virus infection, two similar patient, special environment of their workplace, chemical pneumonitis should be considered. And the follow-up of outpatient, they tell the question that factory find. The ventilation equipment in had fault in these days, leading to the exposure of sodium ibandronate. Besides, only two persons stayed in the room at that time, then they appeared similar situation. The fact confirmed the diagnosis of chemical pneumonitis due to sodium ibandronate.

Sodium ibandronate, a nitrogen-containing bisphosphonate, inhibits bone resorption by osteoclasts (3). It is highly soluble in water and virtually insoluble in organic solvents (4). It is utilized in the treatment of postmenopausal osteoporosis. From the instructions of sodium ibandronate, the side effects include influenza like illness in respiratory system. From Globally Harmonized System of Classification and Labeling of Chemicals (5), sodium ibandronate have health hazard of H332 (Harmful if inhaled [Warning Acute toxicity, inhalation]) and H335 May cause respiratory irritation [Warning Specific target organ toxicity, single exposure; Respiratory tract irritation]. Maybe a reasonable inference is going on both patients. When the mass of drug was shattered without good ventilation equipment, the reaction is exothermic, the hot powder was exposed to air, and some particles dissolved in the ambient moisture, becoming airborne. Then the ventilation machine does not work. Workers inhale the mist of drug. The use of corticosteroids appears to be notably effective in treating inhalation-induced ibandronate sodium injury in the short term.

Although many oral drugs are reported the side effects about inducing lung injury, inhalation-induced injuries are seldom mentioned. Some documented cases include chemical pneumonitis due to bromine compounds (6), zinc oxide fumes (7), and white phosphorus (8). The effectiveness of corticosteroids post-inhalation exposure has not been extensively studied in randomized controlled trials (9). Early administration of corticosteroids has been shown to reduce airway hyperresponsiveness and inflammation in toxic alkylating agents like melphalan (10), with similar findings in chlorine gas-induced injury (11).

From this accident, we should enhance occupational safety awareness. It is important to take safety measures in environments where pharmaceutical powders may be handled, such as in the pharmaceutical industry. It serves as a reminder for workers and workplace managers to be aware of and prevent potential occupational hazards. Besides, both patients in the cases sought medical attention promptly after the onset of symptoms and received timely diagnosis and treatment. This highlights the importance of swift identification and intervention in suspected inhalation injuries for improving outcomes.

## 5 Conclusion

This report presents the first case of chemical pneumonitis due to inhaled ibandronate sodium. The slightly different CT scan presentations in these two cases indicate that chemical pneumonitis can exhibit diverse imaging features. Corticosteroid therapy should be considered in suspected cases, as it generally yields favorable outcomes. This also showed the importance of occupational safety awareness, seeking medical help quickly and rapid treatment.

## Data Availability

The datasets presented in this article are not readily available because all raw data and code are available upon request. Requests to access the datasets should be directed to Ting Pan, panting@zju.edu.cn.
